# Combination Therapy with STAT3 Inhibitor Enhances SERCA2a-Induced BMPR2 Expression and Inhibits Pulmonary Arterial Hypertension

**DOI:** 10.3390/ijms22179105

**Published:** 2021-08-24

**Authors:** Malik Bisserier, Michael G. Katz, Carlos Bueno-Beti, Agnieszka Brojakowska, Shihong Zhang, Sarah Gubara, Erik Kohlbrenner, Shahood Fazal, Anthony Fargnoli, Peter Dorfmuller, Marc Humbert, Akiko Hata, David A. Goukassian, Yassine Sassi, Lahouaria Hadri

**Affiliations:** 1 Cardiovascular Research Institute, Icahn School of Medicine at Mount Sinai, New York, NY 10029, USA; michael.katz1@mssm.edu (M.G.K.); buebecar@gmail.com (C.B.-B.); agnieszka.brojakowska@mssm.edu (A.B.); Shihong.Zhang@mssm.edu (S.Z.); sarahgubara17@gmail.com (S.G.); elkolbg@gmail.com (E.K.); shahood.fazal@icahn.mssm.edu (S.F.); fargnoli2@gmail.com (A.F.); david.goukassian@mssm.edu (D.A.G.); yassine.sassi@mssm.edu (Y.S.); 2 Department of Pathology, University Hospital of Giessen and Marburg (UKGM), Langhansstrasse 10, 35392 Giessen, Germany; dorfmuller@gmail.com; 3 Assistance Publique-Hôpitaux de Paris (AP-HP), Service de Pneumologie et Soins Intensifs Respiratoires, Centre de Référence de l’Hypertension Pulmonaire, Hôpital Bicêtre, 94270 Le Kremlin-Bicêtre, France; mjc.humbert@gmail.com; 4 Cardiovascular Research Institute, University of California, San Francisco, CA 94143, USA; akiko.hata@ucsf.edu

**Keywords:** gene therapy, pulmonary arterial hypertension, right heart failure, BMPR2, SERCA2a, STAT3

## Abstract

Pulmonary arterial hypertension (PAH) is a devastating lung disease characterized by the progressive obstruction of the distal pulmonary arteries (PA). Structural and functional alteration of pulmonary artery smooth muscle cells (PASMC) and endothelial cells (PAEC) contributes to PA wall remodeling and vascular resistance, which may lead to maladaptive right ventricular (RV) failure and, ultimately, death. Here, we found that decreased expression of sarcoplasmic/endoplasmic reticulum Ca^2+^ ATPase 2a (SERCA2a) in the lung samples of PAH patients was associated with the down-regulation of bone morphogenetic protein receptor type 2 (BMPR2) and the activation of signal transducer and activator of transcription 3 (STAT3). Our results showed that the antiproliferative properties of SERCA2a are mediated through the STAT3/BMPR2 pathway. At the molecular level, transcriptome analysis of PASMCs co-overexpressing SERCA2a and BMPR2 identified STAT3 amongst the most highly regulated transcription factors. Using a specific siRNA and a potent pharmacological STAT3 inhibitor (STAT3i, HJC0152), we found that SERCA2a potentiated BMPR2 expression by repressing STAT3 activity in PASMCs and PAECs. In vivo, we used a validated and efficient model of severe PAH induced by unilateral left pneumonectomy combined with monocrotaline (PNT/MCT) to further evaluate the therapeutic potential of single and combination therapies using adeno-associated virus (AAV) technology and a STAT3i. We found that intratracheal delivery of AAV1 encoding SERCA2 or BMPR2 alone or STAT3i was sufficient to reduce the mean PA pressure and vascular remodeling while improving RV systolic pressures, RV ejection fraction, and cardiac remodeling. Interestingly, we found that combined therapy of AAV1.hSERCA2a with AAV1.hBMPR2 or STAT3i enhanced the beneficial effects of SERCA2a. Finally, we used cardiac magnetic resonance imaging to measure RV function and found that therapies using AAV1.hSERCA2a alone or combined with STAT3i significantly inhibited RV structural and functional changes in PNT/MCT-induced PAH. In conclusion, our study demonstrated that combination therapies using SERCA2a gene transfer with a STAT3 inhibitor could represent a new promising therapeutic alternative to inhibit PAH and to restore BMPR2 expression by limiting STAT3 activity.

## 1. Introduction

Pulmonary arterial hypertension (PAH) is a rare and chronic lung disease characterized by the progressive occlusion of the small pulmonary arteries (PAs) and is associated with structural and functional alteration of pulmonary artery smooth muscle cells (PASMCs) and endothelial cells (PAECs) [1,2]. Pulmonary vascular remodeling is characterized by the enhanced muscularization and neointimal thickening of distal pulmonary arteries with endothelial cell hyperproliferation and plexiform lesion formation [3,4]. If left untreated, it may result in right ventricle (RV) failure and death [5]. The RV is extremely sensitive to changes in afterload, and slight variations in pulmonary pressures can impair RV function and global cardiac performance [6]. RV failure increases the morbidity and mortality of PAH patients [7]. Regarding its clinical classification, WHO Group 1 PAH is divided into subgroups that include heritable (HPAH or familial PAH), and non-hereditary forms including idiopathic (IPAH) and associated PAH (APAH), which are related with a variety of systemic diseases such as interstitial lung disease, connective tissue disease, congenital heart disease, or drug/toxin exposures [8,9]. 

The majority of HPAH or IPAH cases have been associated with a loss-of-function (LOF) germline mutation in the bone morphogenetic protein receptor type 2 (BMPR2) gene or a down-regulation of its expression. BMPR2 is a member of the transforming growth factor-β (TGFβ) superfamily involved in embryonic development, vasculogenesis, cell growth, apoptosis, and fibrosis [10]. Most cases of HPAH (>70%) and some IPAH cases (~20%) caused by mutations in the BMPR2 are associated with a down-regulation of BMPR2 expression and weakened SMAD1/5 signaling, causing major phenotypic abnormalities and vascular lesions that are predominantly in the lungs [4,11,12]. Additionally, previous studies showed that patients carrying a BMPR2 mutation manifested PAH at a younger age, exhibit rapid disease progression with severe phenotypes such as extensive pulmonary vascular remodeling and RV dysfunction and have an increased risk of death [13]. However, the low penetrance of BMPR2 mutations suggests that additional genetic or environmental factors may trigger the development of PAH. Therefore, a comprehensive understanding of the pathophysiology of PA remodeling remains essential for evaluating new gene therapy approaches in PAH. A “second hit”, in addition to the LOF germline mutation, may be required for the clinical manifestation of PAH [14]. 

Beyond heterozygous BMPR2 germline mutations in HPAH, both IPAH and APAH are associated with reduced BMPR2 expression and signaling in the lung vasculature of patients [15]. Brock and colleagues have shown that IL-6 activates STAT3 and inhibits BMPR2 expression by up-regulating miRNA-17/92 in human PAECs [16]. Our group has previously demonstrated that Sarco/Endoplasmic Reticulum Ca^2+^-ATPase pump 2a (SERCA2a), a Ca^2+^ handling protein, inhibits the IL-6/STAT3 pathway in lung fibroblasts [17,18]. Altogether, these findings strongly suggest that SERCA2a may regulate BMPR2 expression in pulmonary vascular cells by blocking STAT3 activity. Additionally, we previously demonstrated that decreased SERCA2a expression is associated with excessive proliferation of PASMCs and pulmonary vascular remodeling in IPAH [19,20,21,22]. In vivo, we found that intratracheal delivery of SERCA2a using AAV1-mediated gene therapy induces specific beneficial effects in pulmonary vascular remodeling and RV function by regulating the STAT3 pathway [19]. Therefore, we hypothesized that the long-term efficacy of combination therapies using AAV1.hSERCA2a with AAV1.BMPR2 or a STAT3 inhibitor (STAT3i, HJC0152) might reverse serious respiratory and hemodynamic alterations as well as RV remodeling while restoring RV function in a severe PAH rat model.

## 2. Results

**Figure 1:**
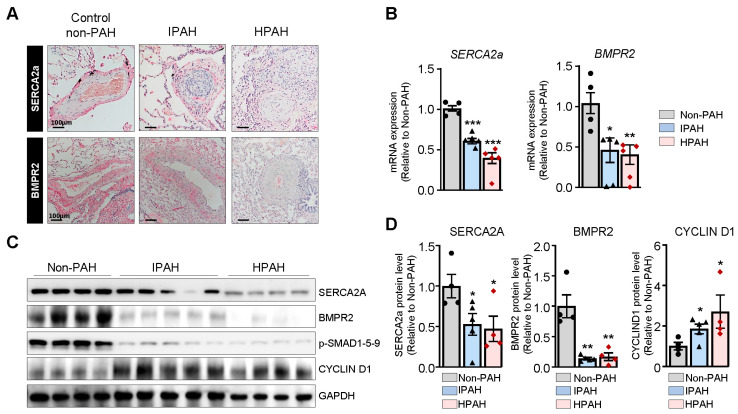
Expression of SERCA2a and BMPR2 in pulmonary vessels from non-PAH, idiopathic, and hereditary PAH patients. (**A**). Representative images of SERCA2a (upper panel) and BMPR2 (lower panel) expression assessed by immunohistochemistry using secondary antibodies conjugated with horseradish peroxidase in lung sections from idiopathic PAH (IPAH, Patient #5 in Supplementary Table S1), hereditary PAH (HPAH, Patient #1 in Supplementary Table S2) patients, and control non-PAH (non-PAH, Patient #1 in Supplementary Table S3). Scale bar: 100 μm. (**B**). SERCA2a and BMPR2 mRNA expression was analyzed by RT-qPCR in lung tissue from control non-PAH (n= 4), IPAH (n = 5), and HPAH (n = 5) patients. (**C**). Representative immunoblots of SERCA2a, BMPR2, Cyclin D1, and phospho-SMAD1-5-9 (p-SMAD1-5-9) assessed through Western blot in lung tissue from control non-PAH (n = 4), IPAH (n = 5), and HPAH (n = 4) patients. GAPDH was used as a loading control. (**D**). Quantification of the indicated proteins normalized to GAPDH. Data are presented as mean ± SEM; * *p* < 0.05, ** *p* < 0.01, *** *p* < 0.001.

SERCA2a expression is markedly decreased in IPAH and HPAH patients. Lung sections from non-PAH, IPAH, and HPAH (carrying BMPR2 mutation p.S301P) patients were analyzed for SERCA2a and BMPR2 expression through immunohistochemistry (Figure 1A). Hypertrophied vessels from patients with HPAH and IPAH demonstrated a marked decrease in SERCA2a and BMPR2 expression compared to non-PAH patients (Figure 1A). Further analysis of the SERCA2a and BMPR2 mRNA and protein levels in the HPAH and IPAH group using RT-qPCR and Western blot showed decreased expression of SERCA2a and BMPR2 in the HPAH group (Figure 1B–D). In contrast, the expression of Cyclin D1 was increased in HPAH lung samples harboring the BMPR2 mutation (Figure 1C,D). 

**Figure 2:**
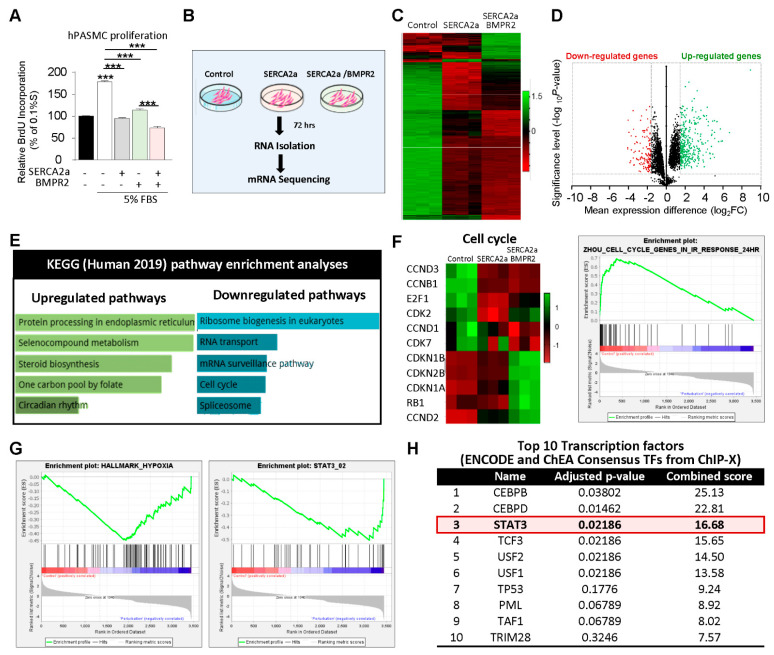
RNA-Seq analysis defines the transcriptome profile of hPASMCs overexpressing SERCA2a and BMPR2 and identifies STAT3 as a potential therapeutic target. (**A**). Proliferation was measured through BrdU assay in hPASMCs overexpressing SERCA2a alone and/or in combination with BMPR2 cultured in 0.1% or 5% FBS for 72 h (n = 5). (**B**). A schematic representation of the RNA sequencing and experimental groups. RNA extraction was performed in hPASMCs overexpressing either an adenovirus encoding β-Galactosidase (control), SERCA2a alone, or in combination with BMPR2 (n = 3). (**C**). Heatmap displaying differential gene expression (DEG) for each sample in the RNA-seq dataset. Each row of the heatmap represents a gene, and each column represents a sample. Down- and up-regulated genes are noted in red and green, respectively. (**D**). Volcano plots showing log2-fold changes and the statistical significance of each gene calculated after DEG analysis. Red points indicate significantly down-regulated genes; green points indicate up-regulated genes. (**E**). RNA-Seq datasets were analyzed using the Kyoto Encyclopedia of Genes and Genomes (KEGG) 2019 database to identify the top 5 up-and down-regulated pathways after SERCA2a and BMPR2 co-overexpression. (**F**). Left panel: heatmap illustrating the expression of genes implicated in the cell cycle. Right panel: Gene Set Enrichment Analysis (GSEA) reveals that cell cycle genes show statistically significant differences in regulation. (**G**). GSEA analysis shows that SERCA2a and BMPR2 overexpression in hPASMCs significantly regulate hypoxia and STAT3-regulated genes. (**H**). Analysis of the RNA-Seq datasets using the ENCODE and ChEA Consensus transcription factors (TFs) from ChIP-X database identified STAT3 among the top 10 regulated TFs. *P*-value and combined score are shown for each TFs. Data are presented as mean ± SEM; *** *p* < 0.001.

Transcriptome analysis by RNA-sequencing in hPASMCs co-overexpressing SERCA2a and BMPR2: We first assessed the effects of SERCA2a in combination with BMPR2 on the proliferation of hPASMCs (Figure 2A). In cultured proliferating cells, SERCA2a overexpression significantly decreased hPASMC proliferation induced by high concentrations of FBS. Likewise, the co-overexpression of BMPR2 significantly enhanced the antiproliferative effects of SERCA2a (Figure 2A), suggesting that a combination of two different therapies may be beneficial. Next, we conducted RNA-seq analysis in hPASMCs overexpressing SERCA2a alone or in combination with BMPR2 to further examine the potential antiproliferative mechanisms of single and combination therapy (Figure 2B). A total of 10,767 differentially expressed genes (DEGs) were identified between the control, SERCA2a, and combined SERCA2a–BMPR2-treated hPASMCs, of which 5361 were significantly up-regulated and 5406 were down-regulated (Figure 2C,D). Pathway enrichment analysis revealed that several up-regulated genes are implicated in protein processing and circadian rhythms (Figure 2E). Several genes involved in the cell cycle checkpoint and proliferation were down-regulated in hPASMCs overexpressing SERCA2a and SERCA2a/BMPR2 (Figure 2F). Gene Set Enrichment Analysis (GSEA) revealed significant inhibition of the hypoxia-regulated (Figure 2G, left panel) and STAT3-target genes (Figure 2G, right panel) after the co-overexpression of SERCA2a/BMPR2 in hPASMCs. We also noticed a significant regulation of gene sets implicated in the endoplasmic unfolded protein response and the epithelial–mesenchymal transition (Supplementary Figure S1). Next, we applied Enrichr to identify the top 10 transcription factors (TFs) regulated in hPASMCs after SERCA2a/BMPR2 co-overexpression using the ENCODE and ChEA consensus TFs from ChIP-X datasets. Our analysis identified STAT3 as one of the top regulated TFs in hPASMCs overexpressing SERCA2a and BMPR2 (Figure 2H).

**Figure 3:**
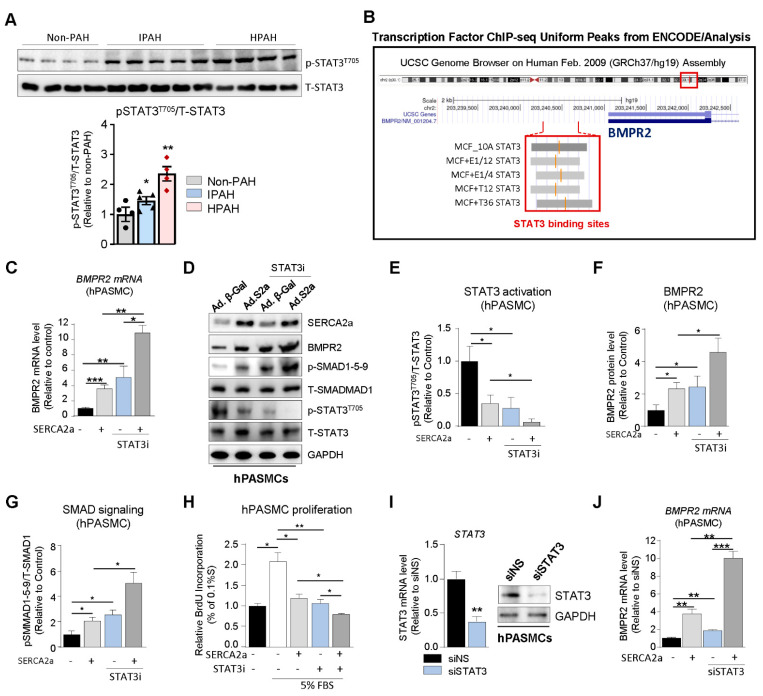
SERCA2a up-regulates BMPR2 expression and downstream SMAD signaling by inhibiting STAT3 activation. (**A**). Upper panel: representative immunoblots of phospho-STAT3^T705^ (p-STAT3^T705^) and Total-STAT3 (T-STAT3) in lung tissue from control non-PAH (n = 4), IPAH (n = 5), and HPAH (n = 4) patients. Total-STAT3 was used as a loading control. Lower panel: the graph represents the quantification of p-STAT3^T705^ normalized to T-STAT3. (**B**). BMPR2 promoter region was analyzed for STAT3 binding sites using Transcription Factor ChIP-seq datasets from ENCODE/Analysis (Feb. 2009 data release). (**C**). hPASMCs were transduced with either SERCA2a or a control vector encoding β-Galactosidase (Ad.βGal) and were treated with potent STAT3 inhibitor HJC0152 (STAT3i) for 48 h (1 µM). BMPR2 mRNA levels were measured by RT-qPCR in the indicated conditions, n = 3. (**D**). hPASMCs were transduced with a control adenovirus (Ad.βGal) or SERCA2a (Ad.S2a) and were treated with STAT3i for 48 h (1 µM). (**E**–**G**). Bar graph represents the quantification of p-STAT3^T705^ (**E**), BMPR2 (**F**), p-SMAD1-5-9 (**G**), and normalized to T-STAT3, GAPDH, and T-SMAD1, respectively, n = 3. (**H**). Proliferation levels were measured through BrdU assay in hPASMCs overexpressing SERCA2a alone for 48 h and/or treated with a potent STAT3 inhibitor (STAT3i, 1 µM) for 72 h in a media containing 0.1% or 5% FBS, n = 4. (**I**). STAT3 mRNA (left) or protein (right) expression was analyzed by RT-qPCR and Western blot, respectively, in hPASMCs transfected either with a non-silencing siRNA (siNS) or a specific siRNA against STAT3 (siSTAT3) for 72 h, n = 3. (**J**). BMPR2 mRNA levels were measured by RT-qPCR in the indicated conditions after 72 h, n = 3. Data are presented as mean ± SEM; * *p* < 0.05, ** *p* < 0.01, *** *p* < 0.001.

SERCA2a up-regulates BMPR2 expression and signaling through a STAT3-dependent mechanism. Since abnormal BMPR2 expression is a pathogenetic hallmark of PAH, we explored the potential role of SERCA2a in the regulation of BMPR2 expression and SMAD-signaling in hPASMCs. First, we assessed the expression of phospho-STAT3^T705^ (p-STAT3^T705^) in HPAH, IPAH, and control non-PAH samples. We found that p-STAT3^T705^ is significantly increased in lung biopsies from HPAH and IPAH patients (Figure 3A). Next, we analyzed publicly available ChIP-sequencing datasets from ENCODE (Feb. 2009 data release) to identify STAT3 binding sites in the BMPR2 promoter. We found binding site enrichment for STAT3 (highlighted in grey) within the BMPR2 promoter (Figure 3B). Given the role of SERCA2a on STAT3 activity [18,19] and the role of STAT3 in PAH as well on BMPR2 expression and downstream targets [16,23,24,25], we tested whether SERCA2a and STAT3 inhibition modulate BMPR2 expression in hPASMCs by using HJC0152 compound, a novel STAT3 inhibitor, and its downstream target genes (c-Myc and Cyclin D1) [26,27,28]. HJC0152 (from here on referred to as STAT3i) is a small molecule compound with potent anti-STAT3 activity and remarkably improved aqueous solubility. It has shown strong anti-tumor activity in several cancer studies, including in glioblastoma, breast and gastric cancer, and head and neck squamous cell carcinomas [26,27,28]. First, our results confirmed that SERCA2a overexpression increased BMPR2 at the mRNA (Figure 3C) and protein level (Figure 3D,F) compared to hPASMCs overexpressing a control adenovirus encoding β-Galactosidase (Ad. β-Gal). Remarkably, we found that SERCA2a overexpression decreased STAT3 phosphorylation (Figure 3D,E, Supplementary Figure S2A). Concomitantly, SERCA2a silencing using a specific shRNA against SERCA2a promotes the phosphorylation of STAT3 at T705 (p-STAT3^T705^) in hPASMCs (Supplementary Figure S2B), confirming that SERCA2a regulates the STAT3 pathway. The pharmacological inhibition of STAT3 (STAT3i) decreased STAT3 activity (Figure 3D,E). Moreover, our results showed that STAT3i treatment significantly abolished p-STAT3^T705^ in hPASMCs overexpressing SERCA2a (Figure 3D,E). Interestingly, STAT3i potentiated SERCA2a-induced BMPR2 expression at the mRNA (Figure 3C) and protein level (Figure 3D,F) and enhanced the subsequent activation of the SMAD1/5/9 pathway (Figure 3D,G). We further evaluated whether STAT3 inhibition, alone or in combination with SERCA2a overexpression, affects the proliferation of hPASMCs. Our results show that STAT3 inhibition decreased the proliferation of hPASMCs and enhanced the antiproliferative properties of SERCA2a (Figure 3H), further suggesting that combination therapies using SERCA2a gene therapy with a STAT3 inhibitor may be beneficial. Consistently, we also found that siRNA-mediated STAT3 knockdown (Figure 3I) significantly enhances BMPR2 mRNA expression in hPASMCs overexpressing SERCA2a compared to control siRNA (Figure 3J). Together, these results suggest that SERCA2a increased BMPR2 expression and signaling by inhibiting STAT3 activity in hPASMCs.

**Figure 4:**
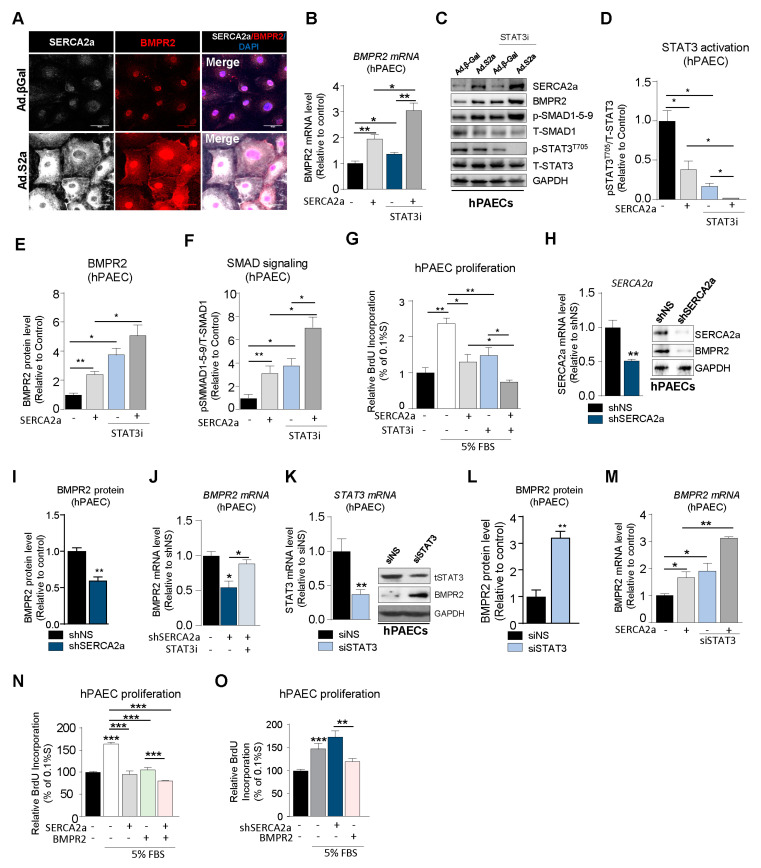
SERCA2a overexpression increases BMPR2 expression in hPAECs. (**A**). SERCA2a (far-red, white color) and BMPR2 (red color) expression was measured by co-immunostaining in hPAECs infected with a control adenovirus encoding β-Galactosidase (Ad.βGal) or SERCA2a (Ad.S2a). Nuclei were counterstained with DAPI (blue). Scale bar: 50 μm. (**B**). BMPR2 mRNA levels were measured by RT-qPCR in hPAECs infected with Ad.S2a or Ad.βGal for 24 hr and were treated with a STAT3 inhibitor (STAT3i; 1 µM) for 48 h, n = 3. (**C**). Representative immunoblots of SERCA2a, BMPR2, phospho-SMAD1-5-9 (p-SMAD-1-5-9), phospho-STAT3^T705^ (p-STAT3^T705^), Total-SMAD1 (T-SMAD1), Total-STAT3 (T-STAT3), and GAPDH in hPAECs in the indicated conditions. (**D**–**F**). Bar graph represents the quantification of p-STAT3^T705^ (**D**), BMPR2 (**E**), p-SMAD1-5-9 (**F**), and normalized to T-STAT3, GAPDH, and T-SMAD1, respectively, n = 3. (**G**). Proliferation levels were measured by BrdU assay in hPAECs overexpressing SERCA2a alone for 48 h and/or were treated with a potent STAT3 inhibitor (STAT3i, 1 µM) for 72 h in a media containing 0.1% or 5% FBS, n = 4. (**H**). SERCA2a mRNA (left) or protein (right) expression was analyzed by RT-qPCR (left) and Western blot (right) in hPAECs overexpressing either a non-silencing shRNA (shNS) or a specific shRNA against SERCA2a (shSERCA2a), n = 3. (**I**). Bar graph represents the quantification of BMPR2 normalized to GAPDH, n = 3. (**J**). BMPR2 mRNA levels were measured by RT-qPCR in hPAECs overexpressing shNS or shSERCA2a alone or in combination with STAT3i (48 h, 1 µM), n = 3. (**K**). STAT3 mRNA or protein expression was analyzed using RT-qPCR (left) and Western blot (right) in hPAECs overexpressing either a non-silencing siRNA (siNS) or a specific siRNA against STAT3 (siSTAT3), n = 3. (**L**). Bar graph represents the quantification of BMPR2 normalized to GAPDH, n = 3. (**M**). BMPR2 mRNA levels were measured by RT-qPCR in hPAECs overexpressing a control vector or SERCA2a alone or in combination with siSTAT3 for 72 h, n = 3. (**N**). Proliferation levels were measured by BrdU assay in hPAECs overexpressing SERCA2a alone and/or BMPR2. Cells were cultured in 0.1% or 5% FBS for 72 h, n = 4. (**O**). Proliferation levels were measured by BrdU assay in hPAECs overexpressing either shSERCA2a alone or with BMPR2. Cells were treated with either 0.1% or 5% FBS for 72 h, n = 4. Data are presented as mean ± SEM; * *p* < 0.05, ** *p* < 0.01, *** *p* < 0.001.

SERCA2a overexpression increases BMPR2 expression in hPAECs. Endothelial dysfunction and structural remodeling of the pulmonary vessels are early features of PAH [29]. Therefore, we further assessed the role of SERCA2a on BMPR2 expression in hPAECs using co-immunostaining and confocal microscopy. Because the infected cells also co-express GFP, we used far-red (white color) to precisely visualize SERCA2a. In hPAECs transduced with a control adenovirus encoding β-Gal (Ad. β-Gal), SERCA2a was detectable at a very low level, and the restoration of SERCA2a expression using an adenovirus (Ad.S2a, white) induced a marked increase in the BMPR2 protein levels (red) (Figure 4A). Similar to hPASMCs, RT-qPCR (Figure 4B) and Western blot (Figure 4C) analysis revealed that SERCA2a overexpression inhibited STAT3 activation (Figure 4C,D, Supplementary Figure S3A) and increased BMPR2 mRNA and protein levels in hPAECs (Figure 4B,C,E). Interestingly, we noticed that the combination with STAT3i potentiated SERCA2a-induced BMPR2 expression and phospho-SMAD1-5-9 signaling by blocking p-STAT3^T705^ (Figure 4C,F). Next, we measured hPAEC proliferation in cells treated with STAT3i alone or in combination with SERCA2a overexpression. Similar to hPASMC, STAT3 inhibition decreased hPAEC proliferation and enhanced the antiproliferative properties of SERCA2a, suggesting that combination therapies using SERCA2a gene therapy with a STAT3 inhibitor may be more efficient (Figure 4G). To further test our hypothesis, the silencing of SERCA2a expression using a specific shRNA (Figure 4H,I, Supplementary Figure S3B) in hPAECs significantly decreased BMPR2 levels (Figure 4I) and potentiated p-STAT3^T705^ (Supplementary Figure S3B). In contrast, SERCA2a depletion in combination with STAT3i (Figure 4J) reversed these effects. Altogether, our gain and loss-of-function approach further confirmed that SERCA2a regulates STAT3 activation and ultimately BMPR2 levels in hPAECs. Similarly, STAT3 knockdown (Figure 4K) increased BMPR2 protein expression (Figure 4K–L). The depletion of STAT3 also potentiates SERCA2a-induced BMPR2 mRNA levels in hPAECs (Figure 4M). Consequently, the combination of SERCA2a and BMPR2 overexpression significantly decreased serum (FBS)-induced hPAEC proliferation (Figure 4N), while SERCA2a depletion (shSERCA2a) reversed this effect (Figure 4O). These findings suggest that SERCA2a limits hPAEC hyperproliferation by restoring BMPR2 expression and downstream signaling through a mechanism involving the STAT3 pathway.

Combination therapy of AAV1.SERCA2a with AAV1.BMPR2 and STAT3 inhibitors in a severe model of PAH: Combination therapy is now considered the standard of care in PAH to target multiple pathogenic pathways [30,31,32]. Having found that the combination of SERCA2a/BMPR2 and SERCA2a/STAT3i inhibited pulmonary vascular cell proliferation and restored BMPR2 in vitro, we evaluated and compared the effects of AAV1.hSERCA2a/AAV1.hBMPR2 and AAV1.hSERCA2a/STAT3i in vivo on pulmonary hemodynamics and vascular remodeling in the severe PAH model induced by unilateral left pneumonectomy combined with monocrotaline (PNT/MCT). The monocrotaline (MCT) model is limited in terms of achieving more advanced disease status in the form of plexiform lesions as well as undesirable toxicity effects on other organ systems. To evaluate the AAV1.hSERCA2a-mediated effects in severe PAH, we used the PNT/MCT model in rats [33]. This model creates a stable, reproducible, and faster rate of PAH progression within 6 weeks with grade 3-4 lesions [33] (Supplementary Figure S4) and significantly increased pulmonary blood flow and pulmonary vascular remodeling with higher RV dysfunction, which is comparable to severe clinical PAH (Supplementary Figure S5) [33].

**Figure 5:**
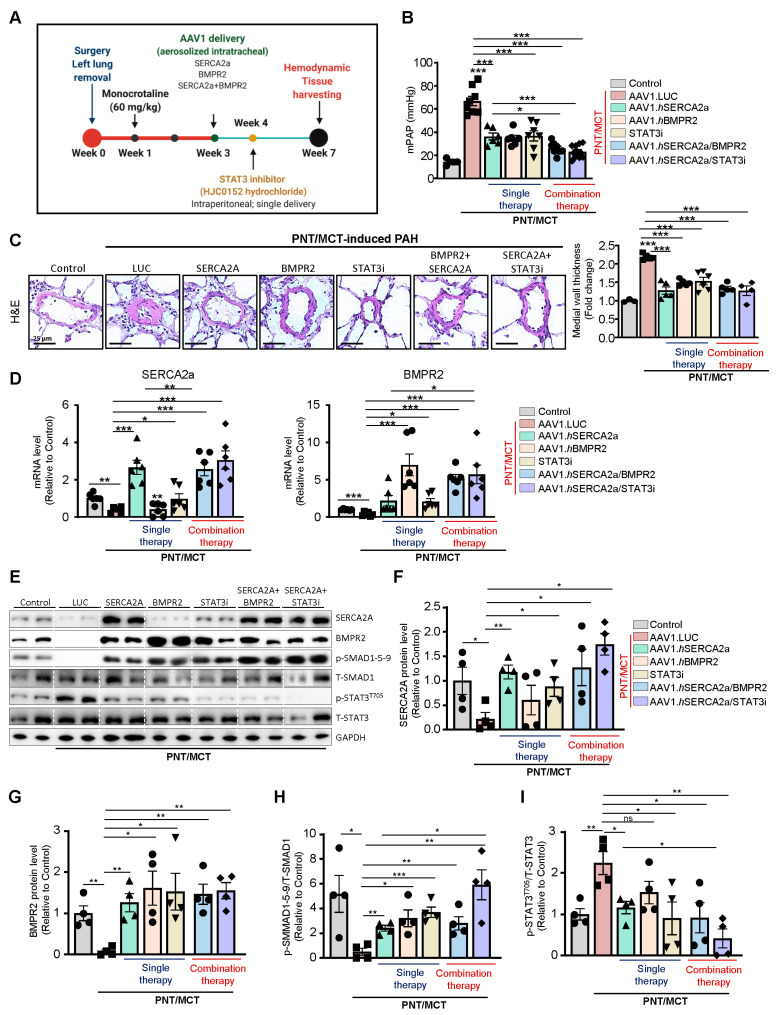
Therapeutic efficacy of single and combination therapy using AAV1-based gene transfer and the STAT3 inhibitor HJC0152 in a severe PAH model. (**A**). Schematic of the experimental design to assess the therapeutic efficacy of single or combination therapy using aerosolized AAV1.LUC, AAV1.*h*SERCA2a, or AAV.*h*BMPR2 gene therapy and a STAT3 inhibitor (STAT3i, HJC0152, intraperitoneal injection) in the rat PAH model induced by unilateral left pneumonectomy combined with MCT (PNT/MCT). Tissues were collected at week 7 for molecular and histology analysis. (**B**). Mean pulmonary artery pressure (mPAP) was assessed in control and PNT/MCT-induced PAH in rats that were either treated with AAV1.Luciferase (AAV1.LUC), AAV1.*h*SERCA2a, AAV1.*h*BMPR2, STAT3i, AAV1.*h*SERCA2a/*h*BMPR2, or AAV1.*h*SERCA2a/STAT3i. (**C**). Representative hematoxylin and eosin-stained lung sections. The graph represents the quantification of the medial thickness. Scale = 25 µm. (**D**). SERCA2a and BMPR2 mRNA expression was assessed in the indicated groups by RT-qPCR, n = 6. (**E**) Lung homogenates were analyzed by Western blot for the indicated proteins, n = 4. Representative Western blots for SERCA2a, phospho-STAT3^T705^ (p-STAT3^T705^), phospho-SMAD1-5-9 (p-SMAD1-5-9), Total-SMAD1 (T-SMAD1), Total-STAT3 (T-STAT3), and GAPDH, n = 4. (**F**–**I**). The bar graph represents the quantification of SERCA2a (**F**) andBMPR2 (**G**) after correction for GAPDH by scanning densitometry. p-SMAD1-5-9 (**H**) and p-STAT3^T705^ (**I**) levels were normalized to T-SMAD1 and T-STAT3, respectively, n = 4. Data are presented as mean ± SEM; * *p* < 0.05, ** *p* < 0.01, *** *p* < 0.001.

To determine the efficacy of monotherapies and combination therapies in the PNT/MCT model, rats received a single subcutaneous injection of MCT (60 mg/kg) one week after removing the left lung. After two weeks, the animals were randomly assigned to 6 groups, including 4 groups with monotherapy that received intratracheal aerosolized AAV1 encoding luciferase (AAV1.LUC) as a control, SERCA2a (AAV1.hSERCA2a), or BMPR2 (AAV1.hBMPR2) or a single treatment of STAT3 inhibitor via intraperitoneal injection (HJC0152, named STAT3i). The two combination therapy groups received AAV1.hSERCA2a with AAV1.hBMPR2 or AAV1.hSERCA2a with STAT3i. The sham rats that were injected with a vehicle only served as controls (Figure 5A). In comparison with the sham control rats, PNT/MCT-PAH rats treated with aerosolized AAV1.LUC exhibited increased mean pulmonary artery pressures (mPAP), which is consistent with PAH-associated phenotypes (Figure 5B). All of the monotherapies, including AAV1.hSERCA2a, AAV1.hBMPR2, or STAT3i, significantly reduced mPAP (Figure 5B). Both combination therapies AAV1.hSERCA2a/AAV1.hBMPR2 and AAV1.hSERCA2a/STAT3i successfully potentiated this reduction and led to a stronger decrease in mPAP in PNT/MCT-PAH rats (Figure 5B). The monotherapies or combination therapies also inhibited pulmonary vascular remodeling (Figure 5C). However, we did not see significant changes in vascular remodeling between the single and dual therapies.

Next, we evaluated the expression level of SERCA2a and BMPR2 in lung samples from all of the groups. Analysis of the transcript levels by qPCR validated the down-regulation of SERCA2a and BMPR2 in the severe PAH model induced by PNT/MCT and confirmed the transduction efficiency of AAV1.hSERCA2a and AAV1.hBMPR2 (Figure 5D). As expected, AAV1.hSERCA2a delivery restored SERCA2a mRNA levels (Figure 5D, left panel) in rats treated with AAV1.hSERCA2a alone or in combination with BMPR2 or STAT3i. Surprisingly, we also noticed that STAT3i monotherapy significantly increased SERCA2a mRNA levels in the PNT/MCT-induced PAH model, compared to PNT/MCT-induced PAH animals treated with AAV.LUC (Figure 5D, left panel). Similarly, BMPR2 mRNA expression was significantly increased after AAV1.hBMPR2 delivery (Figure 5D, right panel). We also found that PNT/MCT-rats treated with AAV1.hSERCA2, AAV1.hBMPR2, or STAT3i monotherapies showed lower IL-6 transcript levels (Supplementary Figure S6). Combination therapy with AAV1.hSERCA2a/STAT3i resulted in more significant reductions in IL-6 levels than monotherapy (Supplementary Figure S6). In addition, the immunoblot analysis further validated our qPCR results and confirmed the transduction efficiency after SERCA2a and BMPR2 gene transfer, as demonstrated by increased SERCA2a and BMPR2 protein levels (Figure 5E–G) and its downstream effector pSMAD1/5/9 (Figure 5E,H). We found that the pharmacological inhibition of STAT3 decreased p-STAT3^T705^ (Figure 5E,I) and restored BMPR2 expression (Figure 5G), confirming our in vitro studies. Surprisingly, STAT3i potentiated SERCA2a expression, suggesting a positive feedback mechanism on SERCA2a expression (Figure 5E,F). Collectively, our results indicate that AAV1.hSERCA2a gene transfer increased BMPR2 expression by blocking the STAT3 pathway. Combination therapy using AAV1.hSERCA2a and STAT3 inhibitors, such as HJC0152, enhanced SERCA2a-mediated effects to inhibit PAH by attenuating pulmonary vascular remodeling and pressure. However, the molecular mechanisms by which SERCA2a expression is dysregulated in PAH remain to be further investigated.

**Figure 6:**
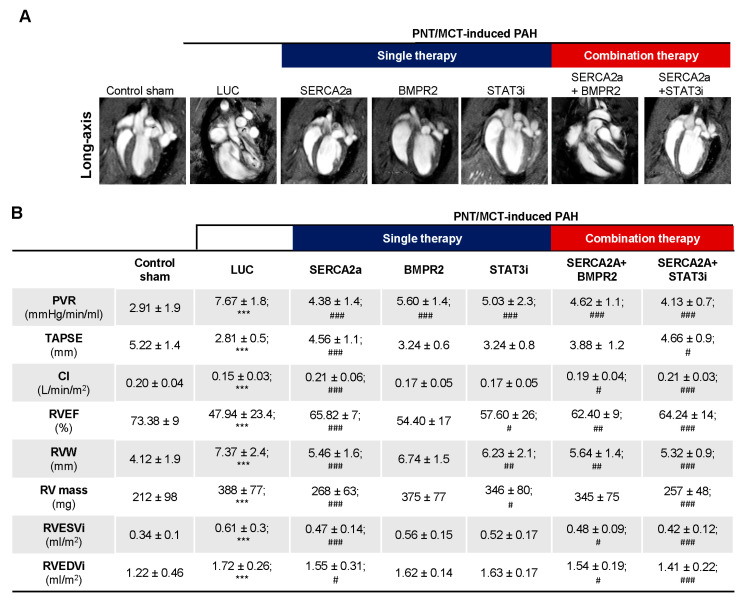
Cardiac magnetic resonance (cMRI) imaging of PNT/MCT-PAH rats after single and combination therapies. (**A**). Representative images of cMRI pictures in long-axis are shown in the indicated conditions. (**B**). Cardiac hemodynamics were measured by cMRI in control (Sham, n = 8) and PNT/MCT-PAH rats treated with aerosolized AAV1.LUC (n = 7), AAV1.*h*SERCA2a (n = 11), AAV1.*h*BMPR2 (n = 9), or intraperitoneally injected with a STAT3i alone (n = 6), or in combination as follows: AAV1.*h*SERCA2a/AAV.*h*BMPR2 (n = 5) or AAV1.*h*SERCA2a/STAT3i (n = 5). PVR: pulmonary vascular resistance; TAPSE: tricuspid annular plane systolic excursion; RVEF: right ventricular ejection fraction; RVW: right ventricle width; RV mass: right ventricle mass; RVESVi: right ventricular end-systolic volume index; RVEDVi: right ventricular end-diastolic volume index; CI: cardiac index. *** *p* < 0.001 vs. Sham. # *p* < 0.05 vs. LUC, ## *p*< 0.01 vs. LUC, ### *p* < 0.001 vs. LUC.

The combination therapy improved RV adaptation and function. The assessment of RV function appears to be a more accurate index of RV performance status. Cardiac magnetic resonance imaging (cMRI), the gold standard for cardiac mass, volumes, and function, was used at different time points to follow the PNT/MCT animals. cMRI was performed at baseline before PNT, and at 3 and 6 weeks post PNT to determine the cardiac morphology and function as shown in Supplementary Figure S5. We observed a gradual decline in RV ejection fraction (RVEF) from baseline to 6 weeks (Supplementary Figure S5A) with increased end-systolic volume (RVESV) and mPAP over time (Supplementary Figure S5B,C) along with structural changes (Supplementary Figure S5D–F). Moreover, representative cMRI images (Figure 6A) show the evolution of PNT/MCT-induced PAH on RV structure in sham controls, PNT/MCT animals treated with AAV1.LUC, AAV1.hSERCA2a, AAV1. hBMPR2, and STAT3i, and the combination treatment AAV1.hSERCA2a/AAV1.hBMPR2 and AAV1.SERCA2a/STAT3i. As expected, we found statistically significant reductions in pulmonary vascular resistance (PVR) after AAV1.hSERCA2a, AAV1.hBMPR2, and STAT3i monotherapy, but the PVR changes were significantly reduced in the combined AAV1.hSERCA2a/STAT3i group (4.13 ± 0.7 vs. 7.67 ± 1.8 in AAV1.LUC, *p* < 0.0001) (Figure 6B). Importantly, the decrease in PVR was accompanied by the normalization of the parameters of RV function such as tricuspid annular plane systolic excursion (TAPSE), cardiac index (CI) in the AAV1.hSERCA2a (TAPSE: 4.56 ± 1.1 vs. 2.81 ± 0.5 in AAV1.LUC, *p* < 0.0001; CI: 0.21 ± 0.06 vs. 0.15 ± 0.03 in AAV1.LUC, *p* < 0.0001), and AAV1.hSERCA2a/STAT3i-treated animals (TAPSE: 4.66 ± 0.9 vs. 2.81 ± 0.5 in AAV1.LUC, *p* < 0.0001; CI: 0.21 ± 0.03 vs. 0.15 ± 0.03 in AAV1.LUC, *p* < 0.0001) (Figure 6B). This resulted in a significant reduction in RV volumes and improvement in RVEF, which are both associated with better survival [34]. In accordance with our previous results and RV functional parameters, the RV mass in the AAV1.hSERCA2 (268 ± 63 vs. 388 ± 77 in AAV1.LUC, *p* < 0.0001) and AAV1.hSERCA2a/STAT3i-treated animals (257 ± 48 vs. 388 ± 77 in AAV1.LUC, *p* < 0.0001) were significantly lower compared to the AAV1.LUC control group (Figure 6B). We also observed a significant reduction in the RV width (RVW) dimensions and the RV end-systolic volume (RVESVi) while the indexed RV end-diastolic volume (RVEDVi) was unchanged (Figure 6B). We only observed significant improvement in RV mass after AAV1.hSERCA2a monotherapy or combination therapy with STAT3i (Figure 6B). Taken together, our findings show that AAV1.hSERCA2a combination therapy with STAT3i enhanced SERCA2a-mediated beneficial effects on RV remodeling and function.

**Figure 7:**
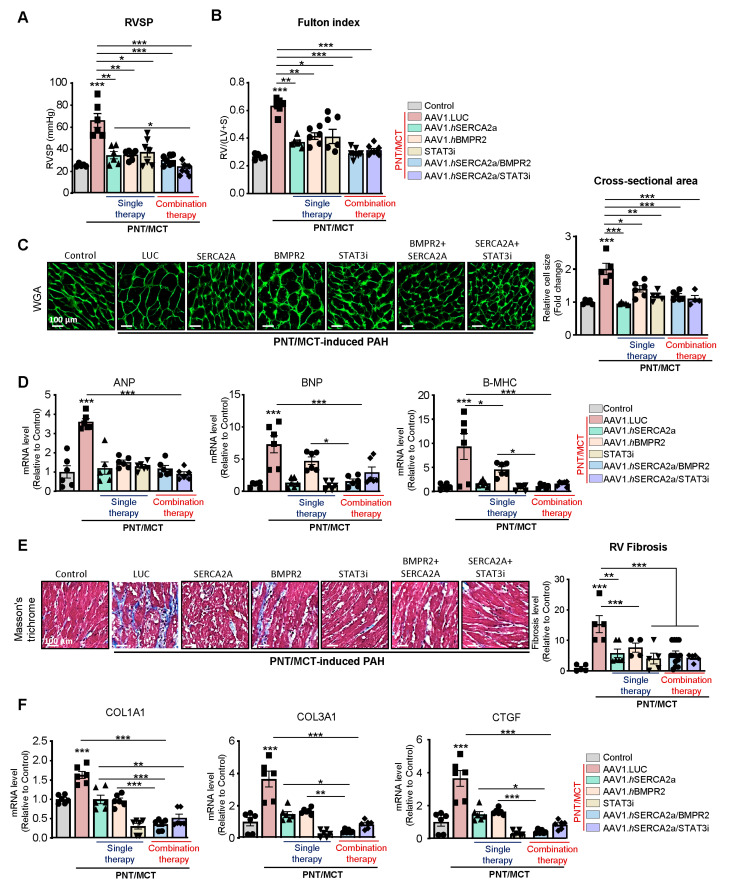
Effects of the combination therapy on RVSP and RV remodeling. (**A**,**B**). Right ventricular systolic pressure (RVSP) (**A**) and Fulton index (RV/(LV + S)) (**B**) were assessed in control sham rats and PNT/MCT-rats treated with either AAV1.LUC (control, LUC), AAV1.*h*SERCA2a, AAV1.*h*BMPR2, or STAT3i alone or in combination as follows: AAV1.*h*SERCA2a/BMPR2 or AAV1.*h*SERCA2a/STAT3i, n = 5–7. (**C**) RV sections were stained with fluorescence-tagged wheat germ agglutinin to examine the RV cardiomyocyte cross-sectional area (left). The bar graph represents the quantification of cardiomyocyte size, n = 4–6. Scale bar = 100 µm. (**D**) Cardiac hypertrophy-related transcript expression levels (ANP, BNP, β-MHC) in the indicated groups assessed by RT-qPCR, n = 6. (**E**) Representative Masson’s trichrome stained heart sections (left). The bar graph represents fibrosis quantification, n = 6. Scale bar = 100 µm. (**F**) Fibrosis-related transcript expression levels (COL1A1, COL3A1, CTGF) in the indicated groups assessed by RT-qPCR, n = 6. Data are presented as mean ± SEM; * *p* < 0.05, ** *p* < 0.01, *** *p* < 0.001.

Effects of the combination therapy on RVSP and RV remodeling: RV enlargement can predict mortality in patients with pulmonary disease and PAH [35]. Right heart catheterization showed a significant decrease in RV systolic pressure (RVSP) with the AAV1.hSERCA2a, AAV1.hBMPR2, and STAT3i monotherapies (Figure 7A). The combined therapies, specifically AAV1.hSERCA2a with the STAT3 inhibitor, significantly reduced RVSP (Figure 7A). RV hypertrophy, assessed by the Fulton index, was also reduced in all of the treated animals (Figure 7B). Subsequently, the RV cardiomyocyte cross-section (Figure 7C) and the RV hypertrophy markers (Figure 7D) were both significantly reduced in AAV1.hSERCA2a alone or in combination with AAV1.hBMPR2 or STAT3i. Interestingly, we also found that RV fibrosis and profibrotic markers were significantly reduced in the AAV1.hSERCA2a-treated animals in single or combination therapies with AAV1.hBMPR2 or STAT3i (Figure 7E,F). Altogether, intratracheal instillation of both combination therapies AAV1.hSERCA2a/AAV1.hBMPR2 and AAV1.hSERCA2/STAT3i is associated with considerable hemodynamic improvement and the inhibition of RV remodeling in severe PAH induced by PNT/MCT.

## 3. Discussion

**Figure 8:**
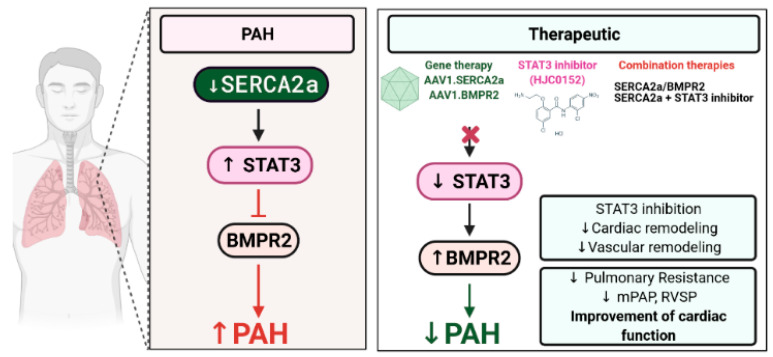
Schematic representation of the molecular pathway by which SERCA2a regulates BMPR2 in pulmonary vascular cells and inhibits PAH. Combination therapy using the STAT3 inhibitor HJC0152 enhanced SERCA2a-mediated effects to attenuate pulmonary vascular remodeling and restore RV function. Created with BioRender.com.

This study provides the evidence for the first time that SERCA2a regulates BMPR2 expression through STAT3 activity inhibition. We found that SERCA2a is down-regulated in lung samples from patients with HPAH and in vitro using cultured proliferating hPASMCs and hPAECs. Herein, we demonstrated that SERCA2a overexpression inhibits the growth of hPASMCs and hPAECs by up-regulating BMPR2 expression and signaling through a mechanism involving the inhibition of the STAT3 activity (Figure 8). In vivo, the combination therapies AAV1.*h*SERCA2a/AAV1.*h*BMPR2 and AAV1.*h*SERCA2a/STAT3i significantly decreased mPAP in the PNT/MCT PAH model in rats. The monotherapies or combination therapies also strongly reduced the severity of PAH by attenuating pulmonary vascular resistance and remodeling, resulting in significant RV function and remodeling improvement.

Previous studies from Reynolds and colleagues showed that the intratracheal delivery of aerosolized adenoviral BMPR2 gene transfer reduced vascular muscularization and RV hypertrophy in the MCT-induced PAH model and in hypoxia-induced PH [36,37]. Similarly, Harper et al. showed that adenoviral gene delivery of BMPR2 inhibited MCT-induced PAH by reducing RV hypertrophy, RVSP, and mean PAP [38]. However, another study conducted by McMurtry and collaborators showed contradicting results in which nebulized adenovirus encoding for human BMPR2 (Ad.hBMPR2) did not improve MCT-induced PAH in rats [39]. Despite the high transduction efficiency, they found no improvement in PAP, PVR, cardiac index, or RV hypertrophy [39]. Vector immunogenicity of adenovirus remains a significant limitation for their use in gene therapy as adenovirus vector-induced innate immune responses may counteract the beneficial effect mediated by transgene overexpression. The recent development of several recombinant AAV serotypes has significantly improved tissue tropism and improved transduction efficiency in smooth muscle cells and endothelial cells while reducing the vector immunogenicity. For example, our group previously showed that SERCA2a gene therapy using AAV vectors reduced pulmonary vascular remodeling and RV load in small and large animal PAH models, including MCT-induced PAH [12,19,20,21,22]. AAV vectors are becoming a promising approach to treating various cardiovascular diseases [18,19,40,41,42,43,44] and have rapidly advanced due to their limited pathogenicity, their ability to transfect both dividing and non-dividing cells, low host immune response, and long-term expression. In PAH, AAV1-based treatment combining SERCA2a and BMPR2 offers long-term and stable expression in the pulmonary vasculature. Additionally, it provides complementary mechanisms of action by targeting multiple pathways.

Our study showed that the co-restoration of SERCA2a and BMPR2 expression reduces PVR and mPAP, thereby inhibiting RV pressure overload and preventing RV failure. Moreover, combination therapies using gene transfer with small compound inhibitors enhance the beneficial properties of each monotherapy by inhibiting the hyperproliferation phenotype of the pulmonary vascular cells while decreasing the vasoconstriction in severe PAH and restoring RV function. By co-targeting multiple candidates involved in the onset of PAH, combination therapies using the gene transfer technology with small compound inhibitors provide a new panel of possible therapeutic interventions for treating severe PAH.

Similar to other STAT proteins, STAT3 is activated when cells are exposed to cytokines and growth factors. After activation, STAT3 translocates into the nucleus and binds to the target-specific sequence gene promoters to regulate gene transcription. In both human and experimental PAH models, many studies have shown that STAT3 hyperactivation modulates the expression of a broad range of proteins and transcription factors (i.e., NFAT, KLFs, and HIF1-α) that are implicated in PAH pathogenesis [23,44,45,46]. Importantly, Brock and colleagues demonstrated that IL-6 stimulation repressed BMPR2 expression by promoting STAT3 binding to the promoter region of the miR-17/92 gene in hPAECs [16]. We previously found that SERCA2a overexpression inhibits IL6/STAT3 signaling in vivo using the bleomycin-induced pulmonary fibrosis model and in vitro in pulmonary fibroblast isolated from healthy donors [17,18]. In this study, we showed that SERCA2a alone or in combination with BMPR2 or STAT3i reduced IL-6 mRNA levels. Additionally, we identified the enrichment of STAT3 binding sites in the BMPR2 promoter region, suggesting that STAT3 may directly target BMPR2 and subsequently PA vascular remodeling. Finally, SERCA2a or STAT3i restores BMPR2 expression and downstream SMAD signaling in vitro and in vivo. Several STAT3 inhibitors are already in early phase clinical trials for the treatment of diverse malignancies [25,26]. Given the proven role of the STAT3 pathway in PAH, our study suggests that drug repurposing of clinically approved drugs against STAT3 may offer a valuable strategy in rapidly delivering new PAH therapeutics into the clinic.

## 4. Materials and Methods

### 4.1. Human Lung Tissue Samples

Lung tissue specimens were obtained from patients with idiopathic PAH (no BMPR2a mutation) or heritable PAH (BMPR2a mutation) (Supplementary Tables S1 and S2) at the time of lung transplantation and controls were collected from patients without PAH (Supplementary Table S3) before thoracic surgery (lobectomy or pneumonectomy for localized lung cancer). Echocardiography was performed in the control patients to rule out pulmonary hypertension. The samples were de-identified and archived. These samples were previously used and published in a prior report from Dr. Hadri et al. [19].

### 4.2. Left Pneumonectomy Combined with Monocrotaline as a Severe Model of PAH in Rats

The procedures described below have been approved by the Institutional Animal Care and Use Committee (IACUC) of the Icahn School of Medicine at Mount Sinai. All rats received human care in compliance with the Mount Sinai “Guide for the Care and Use of Laboratory Animals”. Sprague Dawley rats (250–300 g) were purchased from Charles River. For the left pneumonectomy surgical procedure, the rats were anesthetized with 2–4% isoflurane, intubated via tracheotomy, and mechanically ventilated with 1–2% isoflurane and oxygen flow set at 1 L/min (tidal volume, 0.40 mL; respiratory rate, 70 breaths per minute). After entering the third intercostal space, ligation of the left main bronchus and left main pulmonary artery, the left lung was surgically removed in aseptic conditions, as previously described [33]. Monocrotaline (MCT; 60 mg/kg; Sigma Aldrich, St. Louis, MO, USA) was administered via subcutaneous injection one week after the surgery. Following three weeks post-pneumonectomy, the rats were randomly assigned to receive either a single dose of adeno-associated virus (AAV) serotype 1 encoding Luciferase as a control vector (AAV1.LUC; 3.5 × 10^11^ vg/mL), AAV1 encoding human SERCA2a (AAV1.*h*SERCA2a; 3.5 × 10^11^ vg/mL), either alone or in combination with AAV1 encoding human BMPR2 (AAV1.*h*BMPR2; 3.5 × 10^11^ vg/mL) for four weeks. Treatments were intratracheally (IT) aerosolized in 250 µL using an IA-1C microsprayer (PennCentury, Wyndmoor, PA, USA). A novel potent STAT3 inhibitor, named HJC0152 (MedChem Express), was administered via a single intra-peritoneal injection (1 mL; 7.5 mg/kg) one week after AAV1 delivery. Hemodynamics and morphometric measurements were performed 4 weeks after the mono (AAV1.*h*SERCA2a, AAV1.*h*BMPR2, HJC0152) and combined therapies (AAV1.*h*SERCA2a + *h*BMPR2 or AAV1.*h*SERCA2a + HJC0152) began, and the rats were euthanized for tissue collection.

### 4.3. Cardiac Magnetic Resonance Imaging

Cardiac magnetic resonance imaging (cMRI) was performed at the BioMedical Engineering and Imaging Institute (ISMMS) at three time-points: baseline, three weeks, and six weeks post-pneumonectomy. Hemodynamic parameters were measured by cMRI to assess the therapeutic potential of AAV1.*h*SERCA2a, AAV1.*h*BMPR2, and HJC0152. For cMRI, the rats were transferred on the instrumentation panel bed and were loaded into the Bruker Small Animal 7T MRI unit (BRUKER AXS, Inc., Madison, WI, USA) and maintained with 3% isoflurane via nosecone. Respiration-gated Steady-State Free Precession (SSFP) MRI images was acquired in the short- and long- axis by a dedicated Core Facility technician according to the Bruker’s manufacturer’s protocols. All DICOM images were loaded into the SEGMENT cMRI software and were analyzed by a certified blinded analyst. The short-axis and long-axis CINE series were analyzed for dimensions, volumes, ejection fraction, mass, regional function, and chamber indices via semi-automated contour segmentation. Pulmonary vascular resistance (PVR) was calculated as previously described by Katz et al. [47].

### 4.4. Right Ventricle and Pulmonary Artery Hemodynamic Measurements

Rats were anesthetized with 2–4% isoflurane, intubated via tracheotomy, and mechanically ventilated with 1–2% isoflurane and oxygen flow set at 1 L/min (tidal volume, 0.40 mL; respiratory rate, 70 breaths per minute). The thoracic cavity was opened, and a 1.2 Fr transonic pressure catheter (Transonic Systems Inc., Ithaca, NY, USA) was inserted directly into the right ventricle or the pulmonary artery for direct RV systolic pressure (RVSP) or pulmonary artery pressure (PAP) measurements, as previously described [19,48]. Hemodynamic data were recorded using an ADVantage PV Control Unit (Transonic Systems Inc.). Euthanasia was performed by exsanguination.

### 4.5. Right Ventricular Hypertrophy

The heart was collected immediately after the hemodynamic measurements and were perfused with PBS. Both the atria and the connecting vessels were removed. The RV free wall was separated from the left ventricle and interventricular septum (LVS). The RV and LVS were both individually weighed to calculate the Fulton index to illustrate the RV hypertrophy. The Fulton index is defined by the weight ratio of the RV weight to the LV plus septum weight (RV weight/LV + Septum weight).

### 4.6. Hematoxylin & Eosin, Masson’s Trichrome Stain

Lung tissue was harvested, inflated with PBS/OCT (50:50), and fixed (frozen in −80 °C) in OCT (Tissue Tek, EMS). Sections were cut to 8 μm and were adhered to color frost glass slides (ThermoFisher, Waltham, MA, USA). Lung sections were stained with hematoxylin and eosin (H&E) and Masson’s trichrome (Sigma-Aldrich, St. Louis, MO, USA) and were visualized using light microscopy for histological examination. The medial thickness was measured in distal PAs (<50 µm in diameter) with the following formula: 100 × (external diameter − internal diameter)/external diameter. Collagen deposition was quantified using ImageJ software.

### 4.7. Wheat Germ Agglutinin (WGA) Immunostaining

OCT-embedded RV samples were serially sectioned at 5 µm and were fixed with cold acetone. RV sections were blocked with 5% goat serum for 1 h and were incubated with fluorescence-tagged WGA (Invitrogen) overnight at 4 °C. The sections were then imaged using a Zeiss Observer Z.1 microscope (Carl Zeiss) at 160× magnification. The outlines of cardiac myocytes were traced, and the cardiomyocyte area was calculated using ImageJ software.

### 4.8. Immunohistochemistry

Immunohistochemistry was performed on 7 μm-thick lung sections of paraffin-embedded tissue collected from non-PAH, IPAH, and HPAH patients. After the routine preparation and unmasking of the antigens using a pressure cooker at pH 8 (Electron Microscopy Science, Hatfield, PA, USA), the sections were incubated with horse serum to prevent the non-specific binding of the antibodies or other tissue reagents. The slides were then incubated with a specific rabbit antibody against SERCA2 (1:100; ThermoFisher Scientific) or BMPR2 (1:100, ThermoFisher Scientific). According to the manufacturer’s recommendations, biotinylated horse anti-rabbit, streptavidin-alkaline phosphatase conjugate, and Vector Red Substrate (Vector laboratories, Burlingame, CA, USA) were used for primary antibody detection.

### 4.9. Cell Culture

Human pulmonary artery endothelial cells (hPAECs) were purchased from Lonza, Inc. (Allendale, NJ, USA). hPAECs were cultured in EBM-2 medium supplemented with 5% fetal bovine serum (FBS) supplemented with EGM-2 SingleQuots (Lonza). hPAECs were characterized by immunofluorescence with antibodies specific to vWF/Factor VIII and CD31 (PECAM). Human pulmonary artery smooth muscle cells (hPASMCs) were purchased from Lonza, Inc. (Allendale, NJ, USA) and were cultured in SmBM medium supplemented with 5% FBS and SmGM-2 SingleQuots (Lonza). hPASMCs were characterized by immunofluorescence with antibodies specific to α-smooth muscle actin. Additionally, hPAECs and hPASMCs were further characterized by morphological observation throughout the serial passages and were only used between passages 2 through 7 for this study. Cells were grown in 5% CO_2_ at 37 °C and were passaged at the confluence. All of the cells tested negative for mycoplasma, bacteria, yeast, fungi, HIV-1, hepatitis B, and hepatitis C before use.

### 4.10. Adenovirus and AAV1 Vectors

Adenovirus encoding human SERCA2a (Ad.S2a) and β-galactosidase (Ad-βGal) conjugated with green fluorescence protein (GFP) under the control of the cytomegalovirus promoter were produced as previously described [18,49]. Cells were infected with adenovirus at 100 pfu/cell. The efficiency of infection was assessed by GFP and Western blot.

### 4.11. SDS-PAGE and Immunoblot Analysis

Proteins were extracted using a RIPA lysis buffer (Invitrogen) containing a protease inhibitor cocktail (Roche) and a phosphatase inhibitor cocktail (Sigma-Aldrich). Protein concentration was determined using a bicinchoninic acid (BCA) assay (Sigma-Aldrich) after centrifugation for 20 min at 15,000× *g*. Equal amounts of protein (50 µg) were separated by SDS-polyacrylamide gel electrophoresis (PAGE) and were transferred to polyvinylidene difluoride membranes (Millipore), as previously described [50,51]. The membranes were blocked with either 3% BSA or 5% non-Fat dry milk for 1 h and were hybridized overnight at 4 °C with the primary antibodies listed in Table S2. The membranes were then incubated with the appropriate secondary HRP-conjugated antibody (Cell Signaling). The blots were developed using the SuperSignal West Pico Reagent (Pierce) and were imaged using the Chemidoc MP system (Bio-Rad Laboratories, Hercules, CA, USA). Protein expression was quantified using ImageLab (Bio-Rad Laboratories) and normalized to GAPDH, as previously described [18,46].

### 4.12. Total RNA Isolation, cDNA Preparation, and qRT-PCR Analysis

Cells or the left lobe of each lung were used for total RNA isolation using TRIzol (Invitrogen) and were purified using RNeasymini columns (Qiagen, Hilden, Germany). cDNA was synthesized using the cDNA synthesis kit (Applied Biosystems, Waltham, MA, USA) according to the manufacturer’s instructions. Negative controls without reverse transcriptase were used to verify the absence of genomic DNA contamination. Quantitative PCR (qPCR) was performed using the PerfeCTa SYBR Green FastMix (Quantabio), according to the manufacturer’s instructions. Gene expression was normalized to GAPDH as an internal loading control. The primer sequences are provided in Supplementary Table S4.

### 4.13. siRNA Experiments

Human STAT3-specific siRNA were resuspended in 20 μmol/L (GE Dharmacon) in sterile distilled water. A total of one day after plating, transient transfection experiments were performed using Lipofectamine^®^ 2000 (Invitrogen) using 100 nmol/L of siRNA per well, according to the manufacturer’s instructions. Knockdown efficiency was verified by qPCR and Western blot 72 h post-transfection.

### 4.14. BrdU Cell Proliferation Assay

hPAEC and hPASMC proliferation was measured by 5-Bromo-2′-deoxyuridine (BrdU) incorporation 48 h after indicated treatments using the Cell Proliferation ELISA, BrdU (colorimetric) assay (Roche, Indianapolis, IN, USA) according to the manufacturer’s instructions.

### 4.15. mRNA Sequencing and Transcriptome Data Analysis

For mRNA sequencing, RNA extraction from hPASMCs was performed using a QIAGEN RNeasy Mini Kit (Qiagen), as described by the manufacturer. After quality control assessment (NanoDrop and Agilent 2100 BioAnalyzer), sample integrity, and purity were further evaluated by agarose gel electrophoresis. Only samples with RNA integrity numbers (RIN) above 7, OD260/280: 2, and OD260/230 ≥ 2 were used for RNA-seq. Illumina sequencing was conducted with Novogene (Sacramento, CA, USA) using a state-of-the-art Illumina NovaSeq 6000 platform, a 250–300 bp insert cDNA library with paired-end 150 bp sequencing strategy.

### 4.16. Differential Gene Expression

For samples with biological triplicates, the differential expression analysis of two conditions/groups was performed using the DESeq2 R package [52]. The resulting P values were then adjusted using Benjamini and Hochberg’s approach for controlling the false discovery rate.

### 4.17. Bioinformatics and Data Visualization

Data analysis and visualization were performed using Clustergrammer [53], which is freely available at http://amp.pharm.mssm.edu/clustergrammer/ (accessed on May 2019). Clustergrammer is a web-based tool for visualizing and analyzing high-dimensional data as interactive and shareable hierarchically clustered heatmaps. Clustergrammer enables the intuitive exploration of high-dimensional data and has several optional biology-specific features. Volcano plots. Volcano plots were used to infer the overall distribution of differentially expressed genes. The log2-fold changes and statistical significance of each gene were calculated by performing a differential gene expression analysis. Gene fold changes were transformed using log2 and were displayed on the X-axis; *p*-Values were corrected using the Benjamini–Hochberg method, transformed using −log10, and displayed on the Y-axis. The *p*-value cutoff was set up at 0.05 to display significant genes on the plot. The threshold of the absolute log2-fold changes to indicate differentially expressed genes was set up at 1.5. Heatmap: Before displaying the heatmap, the raw gene counts were normalized using the log CPM method, filtered by selecting the top 2500 genes with the most variable expression, and were finally transformed using the Z-score method.

### 4.18. Statistical Analysis

Results are presented as mean± standard error of the mean (SEM). Data were analyzed using an unpaired t-test for comparisons between means or 1-way analysis of variance with the Bonferroni correction for comparisons between >2 groups. Statistical analysis was performed using GraphPad Prism software, Version 9.0.0 (GraphPad Software, Inc., La Jolla, CA, USA).

## 5. Conclusions

In this study, our goal was to determine the therapeutic impact of SERCA2a-mediated gene transfer and STAT3 inhibition and their effect on BMPR2 expression in pulmonary vascular cells. We demonstrated that SERCA2a increased BMPR2 expression in hPASMCs and hPAECs by blocking the STAT3 pathway. We also found that the antiproliferative properties of SERCA2a are mediated, in part, by the STAT3/BMPR2 axis. Using pneumonectomy combined with monocrotaline (PNT/MCT) as a severe PAH model in rats, we found that combination therapies using aerosolized AAV1.*h*SERCA2a with a potent STAT3 inhibitor (HJC0152) or AAV1.*h*BMPR2: (1) significantly restored BMPR2 expression in the lungs, (2) attenuated the pulmonary vascular remodeling and resistance, and (3) inhibited RV remodeling and significantly restored RV function. Thus, the combination therapy using AAV1.*h*SERCA2a gene therapy with a STAT3 inhibitor could represent a new promising therapeutic option to inhibit PAH and to restore BMPR2 expression in pulmonary vascular cells.

## Data Availability

The data that support the findings of this study are available from the corresponding author upon reasonable request.

## Supplementary Materials

The following are available online at https://www.mdpi.com/article/10.3390/ijms22179105/s1.
