# Supplementary material for: Combination Therapy with STAT3 Inhibitor Enhances SERCA2a-Induced BMPR2 Expression and Inhibits Pulmonary Arterial Hypertension
Source: Int J Mol Sci. 2021 Aug 24;22(17):9105. doi: 10.3390/ijms22179105 (PMC8431626; doi:10.3390/ijms22179105)
Supplement: Supplementary file 1 [file ijms-22-09105-s001.zip › ijms-1348193-supplementary.pdf]

SUPPLEMENTARY MATERIAL

Supplementary Figure S1. Gene Set Enrichment Analysis of RNA-Seq datasets in hPASMC co-overexpressing SERCA2a and BMPR2. Gene Set Enrichment Analysis (GSEA) reveals significant regulation of endoplasmic unfolded protein response and epithelial-mesenchymal transition gene sets.

Supplementary Figure S2. Histological analysis of lung tissue in the severe model of PAH induced unilateral left pneumonectomy combined with a "second hit" of MCT. Using the left pneumonectomy combined with a "second hit" of MCT (PNT/MCT) model, hematoxylin and Eosin in lung tissue show focal pulmonary arteritis and inflammation, concentric medial thickening of the vessel walls, and concentric intimal thickening of vessel walls, resulting in severely constricted vessels. The pneumonectomy combined with MCT model creates a clinically relevant model as revealed by the presence of complex lesions (Grade 2-4).

Supplementary Figure S3. Cardiac magnetic resonance imaging shows significant RV dysfunction and remodeling. Cardiac MRI was performed at baseline and three weeks post-pneumonectomy to confirm PAH severity in PNT/MCT-PAH rats. **A-C.** MRI images were acquired in the short and long axis to determine hemodynamic parameters, including right ventricular ejection fraction (RVEF), right ventricular end-systolic volume (RVESV), and mean pulmonary arterial pressure (mPAP). **D-F.** Long-axis images are shown at baseline, 3 weeks post-pneumonectomy, and 6 weeks post-pneumonectomy in PNT/MCT model of PAH in rats.

Supplementary Figure S4. Monotherapies and combination therapies significantly downregulate IL-6 transcript levels in the PNT/MCT-induced PAH model. IL6 mRNA expression was assessed by RT-qPCR in lung samples from control rats and PNT-MCT-induced PAH rats treated with either AAV1.Luciferase as control (AAV1.LUC), AAV1.hSERCA2a, AAV1.BMPR2, STAT3 inhibitor (STAT3i) alone, or in combination as follow: AAV1.SERCA2a/ BMPR2 or AAV1.SERCA2a/STAT3i.

Supplementary Figure S1

A

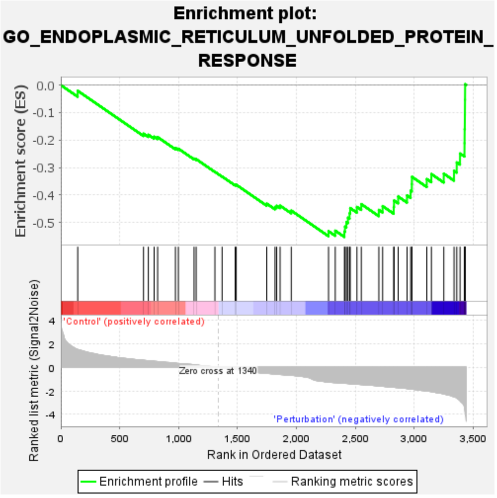

B

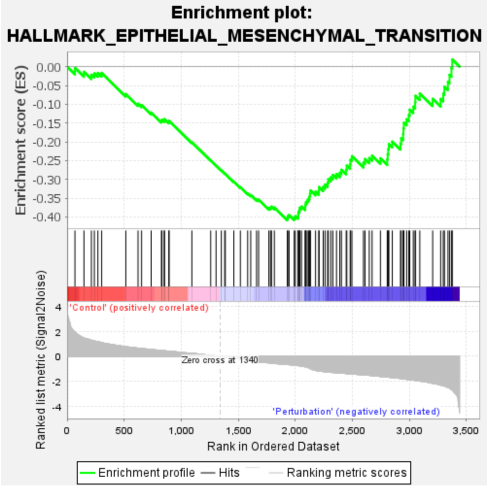

Supplementary Figure S2

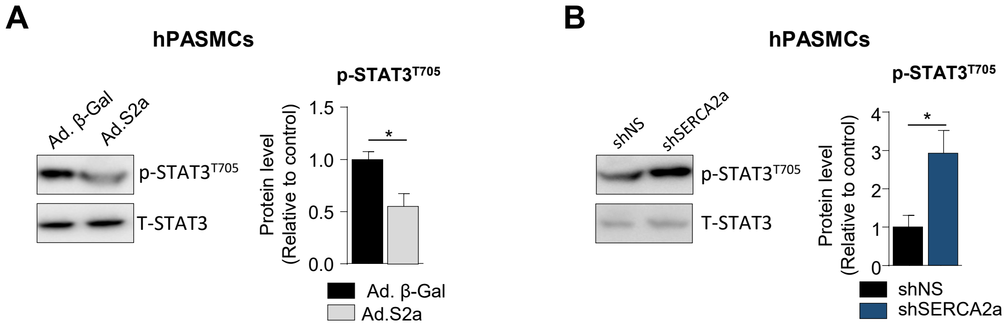

Supplementary Figure S3

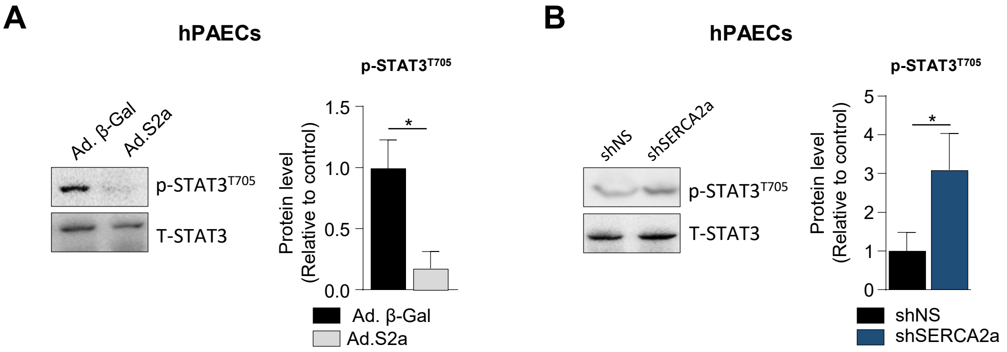

Supplementary Figure S4

PNT/MCT-induced PAH model: Grade 2-4 lesions

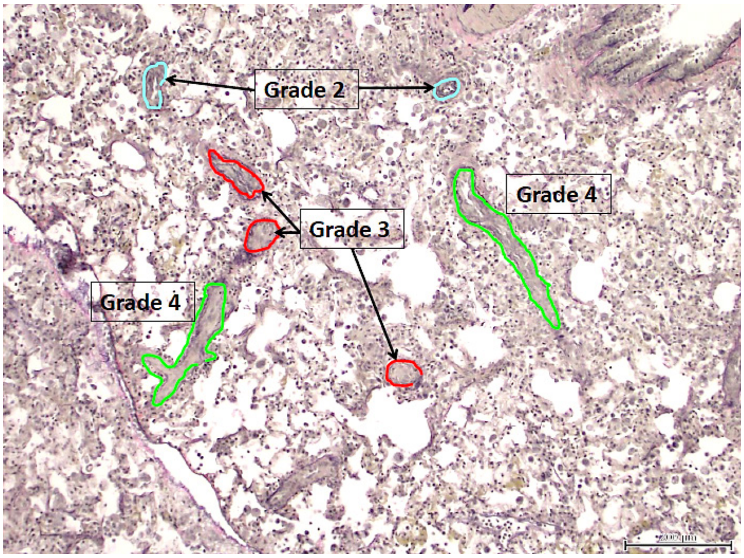

Supplementary Figure S5

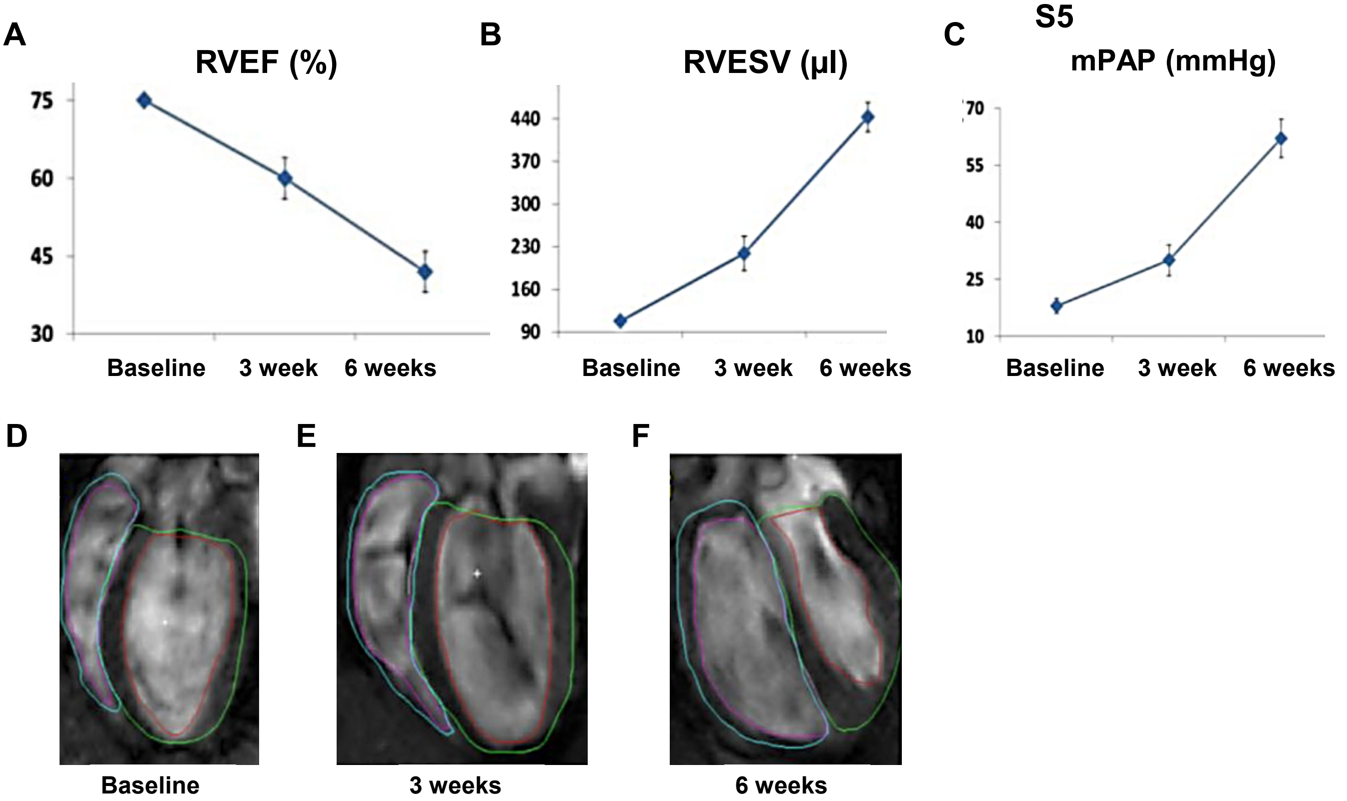

Supplementary Figure S6

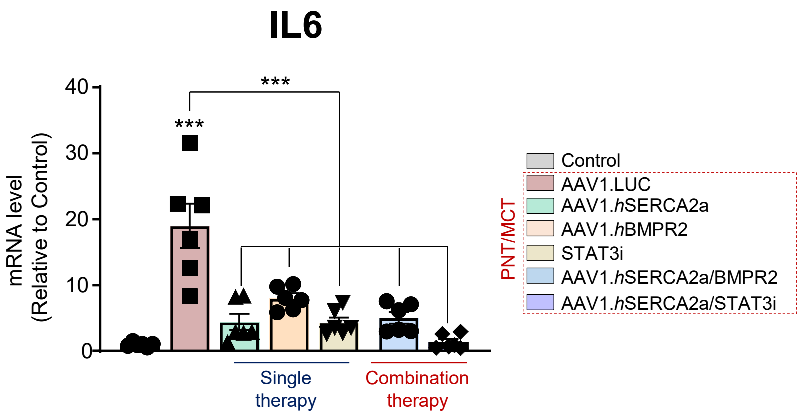

Supplementary Table S1. Idiopathic PAH patients pulmonary hemodynamics pre-lung transplant

	Gender	Diagnosis	Age	Pre-LTx TPR (Wood units)	Pre-LTx mPAP (mmHg)	Pre-LTx CI (l/min/m2)
Patient 1	Woman	IPAH	31	14.5	69	2.45
Patient 2	Man	IPAH	49	13	43	3.23
Patient 3	Woman	IPAH	59	9.4	53	2.85
Patient 4	Woman	IPAH	31	14	61	1.6
Patient 5	Woman	IPAH	18	26.4	100	1.7

Supplementary Table S2. Hereditary PAH patients pulmonary hemodynamics pre-lung transplant

	Gender	Diagnosis	Age	Genetic Status	Mutation		Pre-LTx TPR (Wood units)	Pre-LTx mPAP (mmHg)	Pre-LTx CI (l/min/m2)
Patient 1	Woman	HPAH	22	BMPR2	Exon 7	Substitution 901T>C	29.3	99	1.71
Patient 2	Woman	HPAH	35	BMPR2	Exon 12	Framshift 2522dupCA	11	64	3.25
Patient 3	Woman	HPAH	21	BMPR2	Exon 3	Deletion Exon 3	13.7	55	2.46
Patient 4	Woman	HPAH	26	BMPR2	Exon 10	Deletion Exon 10	6.4	41	4.29
Patient 5	Woman	HPAH	30	BMPR2	Exon 3	Substitution 280T>G	14.1	53	2.15

Supplementary Table S3. Non-PAH control patient diagnosis at the time of surgery

	Gender	Diagnosis
Patient 1	Man	Squamous cell carcinoma
Patient 2	Man	Adenocarcinoma
Patient 3	Man	Adenocarcinoma
Patient 4	Man	Squamous cell carcinoma

Supplementary Table S4. Reagents. Primer sequences for RT-qPCR analysis; clone ID; catalog numbers for shRNAs and siRNA (Open Biosystems); antibodies; source and concentration of chemical inhibitors.

Application	Gene symbol	Species	Forward primer (5′-3′)	Reverse primer (5′-3′)
RT-qPCR	BMPR2	Human	ATCCAGATTATTCTTCCTCCTC	TCACGATGCTGTCAGTATG
		Rat	CTTTGCCCTCCTGCTTCTTGG	CCAAGGTCTTGTTGATACGGGTC
	SERCA2a	Human	AGACCCAAGCTGGCTAGCGTTTA	TTCTTCAGCCGGTAACTCGTTGGA
		Rat	TATGCTGCCAAGACGGTGTT	ACTGGATCAGAGGGCTGGAT
	ANP	Rat	CCCGACCCACGCCAGCATGG	CAACTGCTTTCTGAAAGGGGT
	BNP	Rat	ACAATCCACGATGCAGAAGCT	GGGCCTTGGTCCTTTGAGA
	β-MHC	Rat	ACAGAGGAAGACAGGAAGAACCTAC	CACAAGATCTACTCCTCATTCAG
	COL1A1	Rat	AATGGTGCTCCTGGTATTGC	GGTTCACCACTGTTGCCTTT
	COL3A1	Rat	GAGATGTCTGGAAGCCAGAACCATG	ATCTCCCTTGGGGCCTTGAGGT
	CTGF	Rat	CCCGTTAGCCTCGCCTTGG	GGTACACGGACCCACCGAA
	STAT3	Human	CTGTGGGAAGAATCACGCCT	ACATCCTGAAGGTGCTGCTC
	IL6	Rat	TCCTACCCCAACTTCCAATGCTC	TTGGATGGTCTTGGTCCTTAGCC
	GAPDH	Human	CGACCACTTTGTCAAGCTCA	AGGGGAGATTCAGTGTGGTG
		Rat	TGACAACTCCCTCAAGATTGTCA	GGCATGGACTGTGGTCATGA

shRNAs	Gene symbol	Clone ID	Catalog number
	ATP2a2	TRCN0000038529	RHS3979-201767396

siRNA	Gene symbol	siRNA ID	Catalog number
	STAT3	116558	AM16708

Immunoblotting	Protein symbol	Antibody source	Dilution
	BMPR2	Cell signaling	1:1000
	Cyclin D1	BD Pharmingen	1:1000
	p-SMAD 1-5-9	Cell signaling	1:1000
	T-SMAD 1	Cell signaling	1:1000
	p-STAT3^{T705}	Cell signaling	1:1000
	T-STAT3	Cell signaling	1:2000
	SERCA2a	21st Century Biochemicals, MA, USA	1:2500
	GAPDH	Sigma Aldrich	1:5000

	Compounds	Concentration	Source
**Pharmacological agents**	HJC0152 (STAT3 inhibitor)	1 umol	MedChem Express
